# Evaluating the Power Consumption of Wireless Sensor Network Applications Using Models

**DOI:** 10.3390/s130303473

**Published:** 2013-03-13

**Authors:** Antônio Dâmaso, Davi Freitas, Nelson Rosa, Bruno Silva, Paulo Maciel

**Affiliations:** Centre of Informatics, Federal University of Pernambuco, 50740-540, Recife, PE, Brazil; E-Mails: avld@cin.ufpe.br (A.D.); dsf3@cin.ufpe.br (D.F.); bs@cin.ufpe.br (B.S.); prmm@cin.ufpe.br (P.M.)

**Keywords:** Wireless Sensor Networks, power consumption, simulation, nesC, coloured Petri net

## Abstract

Power consumption is the main concern in developing Wireless Sensor Network (WSN) applications. Consequently, several strategies have been proposed for investigating the power consumption of this kind of application. These strategies can help to predict the WSN lifetime, provide recommendations to application developers and may optimize the energy consumed by the WSN applications. While measurement is a known and precise strategy for power consumption evaluation, it is very costly, tedious and may be unfeasible considering the (usual) large number of WSN nodes. Furthermore, due to the inherent dynamism of WSNs, the instrumentation required by measurement techniques makes difficult their use in several different scenarios. In this context, this paper presents an approach for evaluating the power consumption of WSN applications by using simulation models along with a set of tools to automate the proposed approach. Starting from a programming language code, we automatically generate consumption models used to predict the power consumption of WSN applications. In order to evaluate the proposed approach, we compare the results obtained by using the generated models against ones obtained by measurement.

## 1. Introduction

Wireless Sensor Networks (WSNs) consist of a set of nodes (motes) capable of sensing, storing, processing, and communicating. The critical issue in developing WSN applications is the limited amount of energy usually available in the motes. An application can take years to drain the battery of the sensor node or consume it in a matter of weeks. Therefore, application developers must always adopt best practices in order to reduce the power consumption as much as possible.

The developer shall evaluate the power consumption of applications prior their deployment in the field. By evaluating the power consumption, it is possible to estimate the application's lifetime, be aware of application's power consumption bottleneck, to adopt strategies to increase the network lifetime, to anticipate the time to replace the sensor node (to maintain always-on network) and so on.

The best way to carry out the power consumption evaluation is directly on the physical hardware, by periodically measuring the remaining battery, as was used by [1–4]. This process, however, has several problems such as the need of high financial investment, reproducibility of the environment, inherent dynamism, complexity and size of WSNs (thousands of nodes), and the potential impact of hardware and human failures.

Another way for conducting the power consumption evaluation is by adopting modelling. Such models may be analytically evaluated and/or simulated. Although modelling may provide less accurate results than measuring, it provides the designers the flexibility and agility to evaluate complex scenarios without interfering on the actual environment. Furthermore, evaluating WSN applications at higher level (software level) may have many advantages, but the most prominent is the time required to conduct the analysis. These are key attributes in mature and well defined power-aware design process [5].

In this context, this paper presents an approach for evaluating the power consumption of WSN applications through modelling. A set of basic models has been defined in order to express the power consumption of commands and structures of a programming language (nesC), whilst a translator takes responsibility of generating the respective model (coloured Petri net—CPN [6–8]) for the whole application. Starting from a WSN application written in nesC, its power consumption model is generated and evaluated in such way that enable us to automatically check the impact on the power consumption of every change in the application.

Considering what is being proposed, this paper has the following unique contributions: (1) a fully automated process for evaluating the power consumption of WSN applications, (2) a complete set of reusable CPN consumption models of WSN applications, and (3) a tool that generates power consumption models from WSN application code written in nesC. It is worth observing that nesC is the programming language most used for implementing actual WSN applications, whilst Petri nets has been widely adopted as a powerful modelling notation used to many different purposes and in several application domains [9,10].

The rest of this paper is organized as follows: Section 2 introduces basic concepts necessary to understand the rest of the paper. Next, Section 3 presents the proposed models and tools. Section 4 performs an experimental evaluation of the proposed solution, followed by the presentation of related work in Section 5. Finally, the last section presents conclusions and future work.

## 2. Background

In this section, we describe some basic concepts in order to help to understand the paper's contributions. Initially, we present coloured Petri nets, followed by basic concepts of WSNs. Finally, we conclude this section showing some notions of TinyOS and nesC.

### 2.1. Coloured Petri Nets (CPNs)

Petri nets (PN) [11] are a family of formal techniques used to model a wide range of systems through a graphical notation. Petri nets enables us to check properties about the proposed models, such as reachability, boundedness, liveness and reversibility [9]. Nowadays, there are many extensions (e.g., timed, stochastic, coloured) that have been defined with particular purposes. In common, all of them have four basic elements: places, transitions, arcs and tokens. The places represent the system variables, transitions represent actions (or events) that occur within the system, arcs denote relations between places and transitions and vice versa, and the tokens are the place's values.

CPN [6–8] is an executable model that is both state and action oriented, and combines the capabilities of Petri nets [9,10] with the capabilities of a high-level programming language (CPN ML). CPN ML is a functional programming language based on Standard ML [12] that provides the primitives for the definition of data types. CPN considers the concept of time that allows to capture the delay taken by events in the system. Therefore, CPN can be applied to evaluate performance measures. It worth mentioning that having the capability of programming languages is quite handy for stochastic simulation purposes and for random variation generation, in particular, so fundamental in performance studies.

#### 2.1.1. CPN Tools

**Figure 1.**
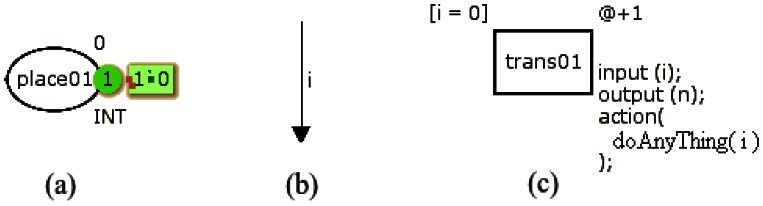
(**a**) place and token, (**b**) arc and (**c**) transition representations in CPN Tools.

CPN Tools [13] is the main tool used to design coloured Petri nets. Each place has a name, a colour set and an initial marking. The name identifies the place, the colour set determines the types of tokens that can be stored, and the place may also have an initial value. This initial value is used to start the CPN model. Figure 1(a) illustrates a place (*place01*) containing one token set to zero. Outside the place there are an initial marking (set to 0) and a colour set (*INT*).

Figure 1(b) shows an arc with a single annotation that is a variable (*i*) of the same type of the input place colour. Figure 1(c) illustrates a transition containing: identification (*trans01*), guard, time and code. The guard ([*i* = *0*]) is a Boolean expression and must be true to enable the transition. When the transition is fired, the time fragment (@+*1*) adds a certain amount of time in the CPN model and the code assigned to the transitions must be executed. The code is divided into three parts: *input*, *output* and *action*. The *action* contains the CPN ML code to perform some activity that processes the input (*input*) and returns a value (*output*).

Hierarchical CPN aims to represent CPN models at different abstraction levels that that may be connected to each other. This feature is very useful for building large models (e.g., to represent an application). In practice, this feature enables us to divide the model into multiple modules, where each module has a CPN. In addition, it allows the reuse of modules, which reduces the size of the model. These independent models (called sub modules) are represented by a substitution transitions in the main module. These modules are connected through places and the connections always involve only two places: one in the sub modules (called *port*) and another in the super modules (called *socket*). A *socket* passes a token to its respective *port*, and *vice-versa*. There are three kinds of ports: input, output, and I/O. An input port receives a token from the socket; an output port sends a token to the super modules and I/O port can both send and receive tokens.

The CPN Tools has an important mechanism to periodically extract information about marking of places and occurring biding elements: Monitor. A monitor may be used to observe, inspect, stop, control or modify the simulation of a CPN model. In practice, the monitor can observe and take appropriate actions based on the observations. There is support for four kinds of monitors, namely breakpoint (to stop a simulation), data collector (to extract numerical data from a net), write-in-files (to update files during simulations) and user-defined (used to any purpose).

State space models, such as Petri nets, aim at automatic analysis and verification of the behavior of systems. The basic analysis methods build the system's state space considering all state transitions (reachability graph). This process can be fully automated. Unfortunately, such methods suffer from a problem that is fundamental that led many to believe that state space methods would never work well enough for large systems. Nevertheless, the great rewards of the state space models have stimulated researchers to undertake studies to lessen the problem [14]. There are many strategies and methods to tackle this problem, among them, it should be highlighted approaches that exploits structural attributes of the model [10,11], reductions in the reachability graph and at the net level [15], exploration of symmetries within the reachability graph [9], the adoption of partial state space generation methods [16,17], and simulation [6,18,19]. Simulation itself may be understood as a partial state space generation methods, since it stops generating new states when a criterion is reached. This particular work extensively uses reductions at the net level and adopts stochastic simulation as the fundamental strategy for conducting the evaluation.

### 2.2. Wireless Sensor Network (WSN)

Wireless Sensor Networks (WSNs) are an emerging technology which consists of the improvement of three technologies [20]: microprocessors, wireless communications and micro-electromechanical systems (MEMs). A WSN is typically formed by thousands of small nodes, with low computational power, few available resources (e.g., memory), a short-range communication and generally non-rechargeable battery. The set of nodes allows the network to be able to perform complex tasks, which may be difficult to be performed by a single node [21]. WSNs have usually a dynamic topology and each node has a type of sensing equipment to analyze a given phenomenon (e.g., temperature, luminosity). Through this device, it can capture the phenomenon occurred, and transmit it to a sink node, responsible for disseminating the data to the observer (or the Internet). Due to the large number of participating nodes in the network, it can be considered as an advantage the short distance between them, using a multi-hop communication (as opposed to using long-range communication) to save energy in data transmission [22].

#### 2.2.1. TinyOS and nesC

TinyOS [23] is an open-source and event-based operating system specially designed to run in devices with low computational power and limited storage as WSN nodes. TinyOS executes in many different motes (e.g., IRIS [24], MICAZ) and provide facilities for the development of applications. TinyOS was developed in nesC [25], an extension to C, optimized to meet the limited storage of sensor nodes.

In terms of programming, an application in nesC is built out of components that are wired together. A component has a specification and an implementation in which the specification defines provided/used interfaces, whilst the implementation consists of the nesC code that implements the specification. In practice, the provided interface characterises the functionality of the component and the used interface defines the functionality required by the component.

An interface in nesC is bidirectional and used to communicate the components. The interface specifies a set of functions named *command* and *events*, in which *commands* must be implemented by the interface's provider, whilst *events* must be implemented by the interface's users. *Task* is another function present in the component, beyond *commands* and *events*. It is a special kind of function that neither returns anything (void) nor has arguments. A task works as an independent locus of control and unlike *events* and *commands*, are posted in a queue and lately executed by a schedule (following a FIFO policy). In this way, interactions between components may be very complex and typically a component registers the interest in some *event* that is signalized when it occurs. For example, a component that invokes a *command* like “read temperature” must implement the *event* “read temperature done” that is executed right after the completion of *command* “read temperature”. In practice, commands typically go “downwards” (from application to closer the hardware) and events call “upwards” (closer to the hardware to the application).

nesC has an execution model consisting of run-to-completion tasks (non pre-empted) and interrupt handlers that are signalled asynchronously by the hardware. The aforementioned scheduler is responsible for executing the tasks in any order and, as the tasks cannot be pre-empted, they are atomic with respect to each other, but are not atomic with respect to interrupt handlers. As a consequence of this execution model, the code of a nesC application may be divided into two parts: synchronous code (SC) and asynchronous code (AC). The SC is only reachable from tasks and includes functions, commands events and tasks. On the other hand, the AC is reachable from at least one interrupt handler.

In terms of programming, nesC has the same set of operators of the language C: math (+, ×, −, /), function call, assignment (=, +=, −=, ×=, /=), byte (<, >, &, |), logical (>, >=, <, <=, ==, !=), cast, and primary expressions (identifiers, constants, string literals, parenthesized expression). The function call has new operators in nesC: *call α* used to invoke a command (*α*); *signal ε*, for call an event (*ε*); and *post Ω*, for invoking a task (Ω). Additionally, the language has statements divided into three categories: selection, iteration and jump statements. Selection statements choose one branch to execute based on the evaluation of an expression (called controlling expression;. Selection statements may have many branches and each branch has a Boolean expression and a body. The selection statement executes the first branch whose evaluation of the controlling expression is true. This kind of statement has two representations (*if-then-else* and *switch*) as shown in the following”.

If (expression) {switch (expression) expression list;{} case 0: expression list;Else if (expression){ case 1: expression list; expression list; …} default: expression list;…}  (**a**)  (**b**)

Iteration statements execute the attached statement (called the body) repeatedly until the controlling expression evaluates to false [26]. It has three statements: *while*, *do-while* and *for*. The controlling expression of a *while* statement is evaluated before each execution of the body. Whilst, the controlling expression of a *do-while* statement is evaluated after each execution of the body. Finally, *for* statement is used to execute many times the body. This statement has three expressions: first expression is associated with the initialization, the second expression is the controlling statement, which is evaluated before of the iteration, and last expression usually specifies the size of increment.

While (exp)dofor (exp, exp, exp){{{expression list;expression list;expression list;}} while (exp);} (**a**) (**b**) (**c**)

The control flow in an iteration statement can be altered by a jump statement. Jump statements cause unconditional transfer of control [26]. It has three statements: *continue*, *break* and *return*. The *continue* statement ignores the instruction subsequent and pass the control to the controlling statement. The *break* statement terminates the switch (only selection statement) or iteration statements, whilst the *return* statement finishes the function.

## 3. CPN Models

**Figure 2.**
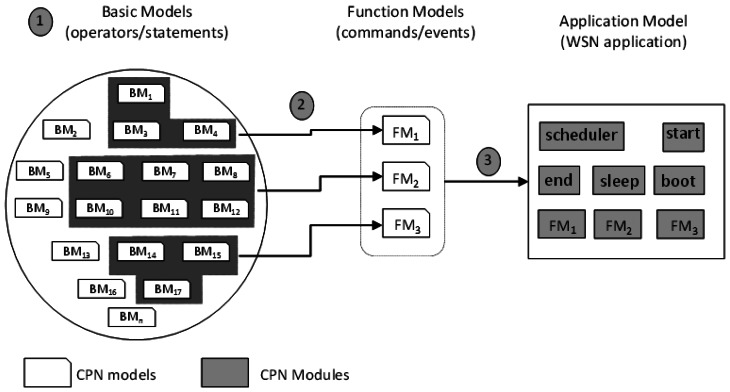
Schematic view of the CPN models definition.

In order to evaluate the power consumption of WSN applications, we have followed three main activities as shown in Figure 2.

Initially, we defined a set of reusable CPN models (referred to “basic models”) to express the power consumption of nesC operators (e.g., assignment) and statements (e.g., *if-then-else*). Each nesC statement/operator is modelled through a basic CPN model and has associated its respective power consumption (obtained by measurement using actual WSN motes). Next, these basic models are composed into a model (referred to “function model”) to express the power consumption of nesC functions (tasks, events and commands). Finally, as a nesC application consists of tasks, commands and events, we compose the function models to express the power consumption of the whole application in what we call “application model”. This model represents a nesC application independent from a particular hardware platform. These models are described in the next subsections.

### 3.1. Basic Models

First step towards the modelling of power consumption of nesC applications is to define CPN models for each statement and operator of nesC. As some of them have the same structure, it is possible to group them into five different groups: operators, calling commands, selection statement, iteration statement and event *receive*. Next subsections present details about these groups.

#### 3.1.1. Operators

**Figure 3.**
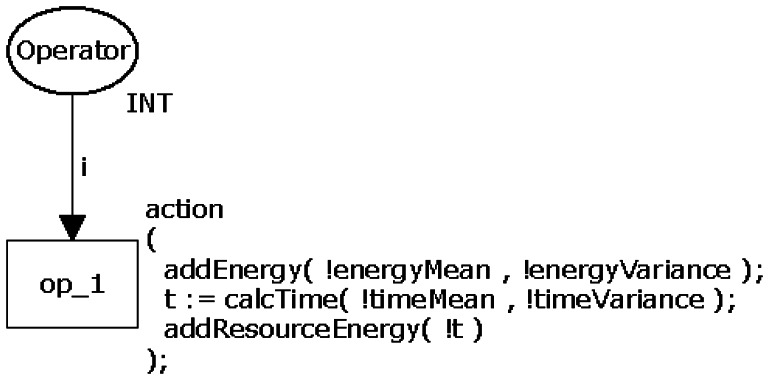
Generic CPN model of the nesC operators.

Due to their similar structures, operators such as arithmetic (+, −, ×, /, and the modulus operator %.), relational (>, >=, <, <=, ==, !=), logical (&& and ‖), increment (++), decrement (−−), bitwise (&, |, ^), ≪, ≫,∼), and assignment (=; have been modelled through a generic CPN model as shown in Figure 3. The transition *op_1* represents the operator and has associated the power consumption calculations.

An action (*action*) is executed when *op_1* is triggered and consists of three calculations performed by the functions addEnergy(), calcTime() and addResourceEnergy(). The function addEnergy() calculates a random value following the normal distribution of the instruction's power consumption at that time using its input parameters (*energyMean* and *energyVariance*). Next, this function updates the power consumption (global variable) of the entire application. By generating a random value, it is possible to define a confidence interval that must contain it. Function calcTime() calculates the time consumed by the operation. Similarly to the previous function, calcTime() also uses the mean and variance to generate a random value to the time. Finally, the function addResourceEnergy() calculates the power consumed by the resources of the mote (radio and LEDs) that may be active while the operation executes (see more details in Section 3.4). It is worth observing that the aforementioned mean and variance of time/power consumption are particular to each operator and are obtained by measurement.

This model has two properties (see Section 2.1): bounded, because place *Operator* does not store tokens; and it has not dead transition, because transition *op_1* will always run.

#### 3.1.2. Selection Statements

The selection statements *if-then-else* and *switch* are represented using the same CPN model as they have branches, and only one of them runs (see Section 2.2.1). As these statements have branches, it is necessary to associate the probability of each branch to be true. The application developer is responsible for manually associating this probability through a C comment. For example, in the following nesC code, the probabilities associated by the application developer to each branch are: 80% (E_SUCESS), 15% (E_ERROR) and 5% (otherwise).


switch (e){ case E_SUCESS: //@0.80  value = v; break; case E_ERROR: //@0.15  value = -1; break; default: //@0.05  value = 0;}

**Figure 4.**
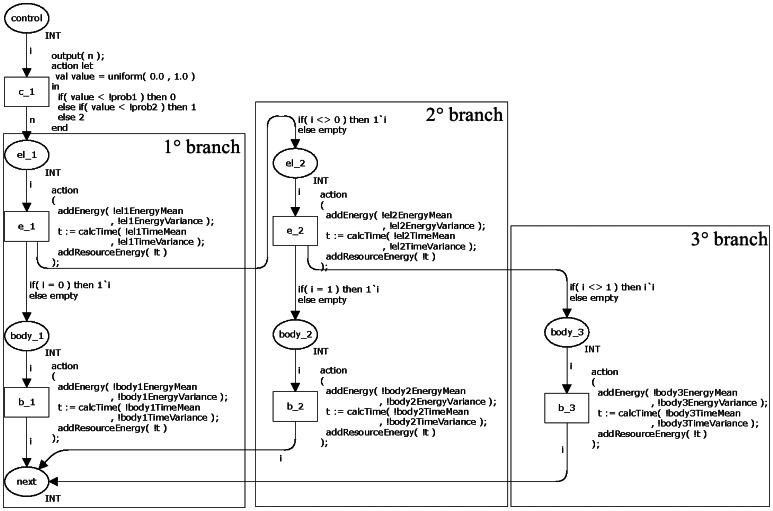
CPN model of the commands *if-then-else* and *switch*.

Figure 4 shows the CPN model for representing the statements *if-the-else* and *switch*. The transition *c1* decides the branch to be executed by generating a random value between 0 and 1 following the uniform distribution. The token value is the decided value by this transition and this value will be used by the subsequent transition.

The places *el_x* (e.g., *el_1* and *el_2*) and *body_y* (e.g., *body_1*, *body_2* and *body_3*) have power consumption calculations related to the each branch. The place *el_x* represents the controlling expression and *body_x* represent the body.

As mentioned in Section 2.2.1, a selection statement executes the branch when its controlling expression returns true. In other words, the controlling expression of a branch consumes energy before deciding if it executes the body (when return true) or passes the control to the next branch (when return false). The transition *e_x* (e.g., *e_1* and *e_2*) represents this behaviour in the CPN model. This transition checks if it runs the body or passes to the next branch. For example, the place *el_1* verifies the token value and move the token to place *body_1* (when the value is equal to 0) or to place *el_2* (when the value is nonzero). The transition *e_2* has a similar behaviour.

This model has similar properties to the previous model when all branches have a probability greater then zero. On the other hand, in the case a branch has associated a probability equal to zero (//@0.0), it means that its body will never be executed. Consequently, the transition associated to this branch (e.g., *b_1*, *b_2* and *b_3 in*Figure 4) becomes a dead transition.

#### 3.1.3. Iteration Statements

Due to their particularities, the loop statements (*while*, *do-while* and *for*) have different CPN models. The statement *while* has a control that (1) checks if an expression is true and (2) then executes the body (in case the Boolean expression is true). When the body terminates, the control checks the expression again. This cycle ends when the expression returns false. Similarly to the command *if-then-else*, the command *while* also has an expression that must be evaluated in order to decide what to do next. Hence, a probability is associated to the control return, *i.e.*, the probability of the loop repeats. Similarly to the selection statements (Section 3.1.2), the application developer must associate a probability (commands *while* and *do-while*) or a number of times the body runs (statement *for*) to the loop control.

**Figure 5.**
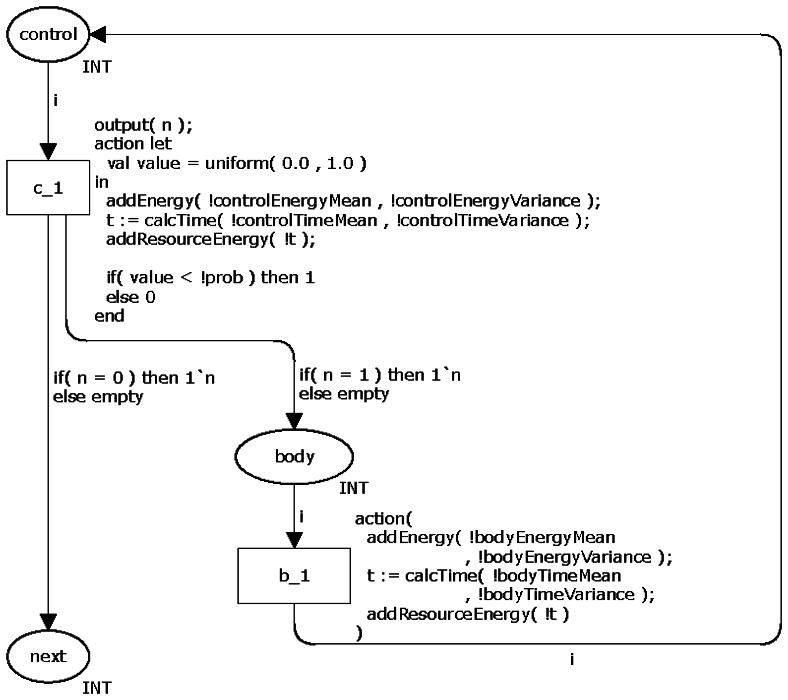
CPN model of the command *while*.

Figure 5 shows the CPN model for representing the command *while*. The transition *c_l* is similar to one adopted in the command *if-then-else* and decides whether the loops repeats (the token moves from place *control* to *body*) or not (token moves to *next*). The place *body* models the command(s) executed in the body. Similarly to the selection statements, this model may have dead transitions when the probability associated to the commands is zero.

Similarly to the previous commands, the power consumption of command *while* is also calculated using the functions addEnergy(), calcTime() and addResourceEnergy().

**Figure 6.**
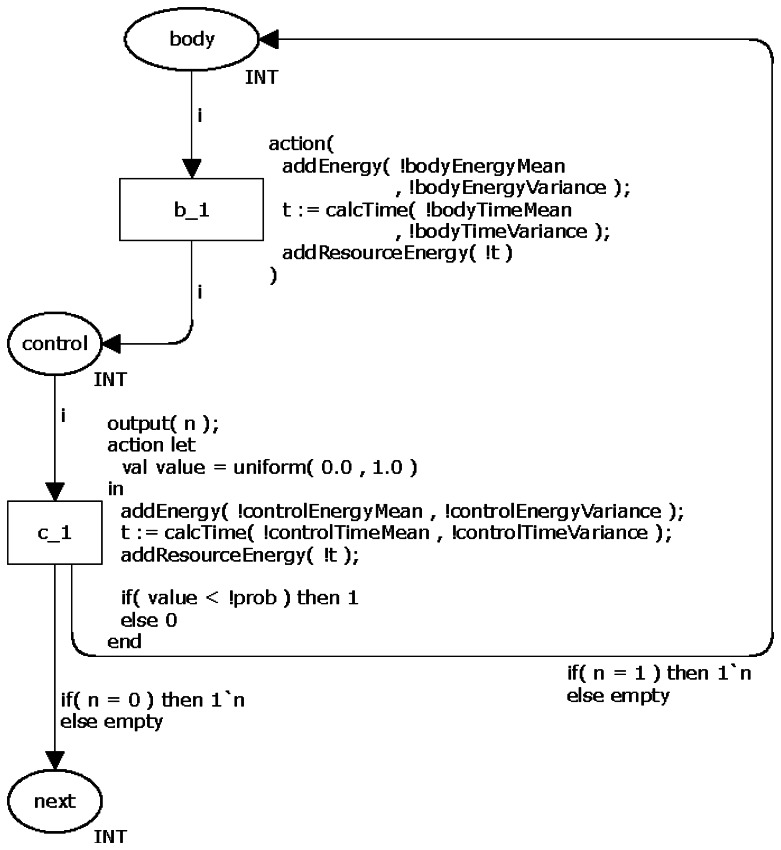
CPN model of the command *do-while*.

The CPN model for representing the command *do-while* is very similar to the command *while*. The only and fundamental difference between them is that the *do-while* ensures that the body will be executed at least once. This feature means that this model has not dead transitions. Figure 6 depicts the proposed CPN model.

Finally, the last loop command, namely *for*, consists of four parts: initialization, control, step and body. The first three ones may be modelled as shown in Section 3.1.1, *i.e.*, by using the assignment, logical and increment/decrement operators. The body part is similar to those presented in the previous loop commands.

**Figure 7.**
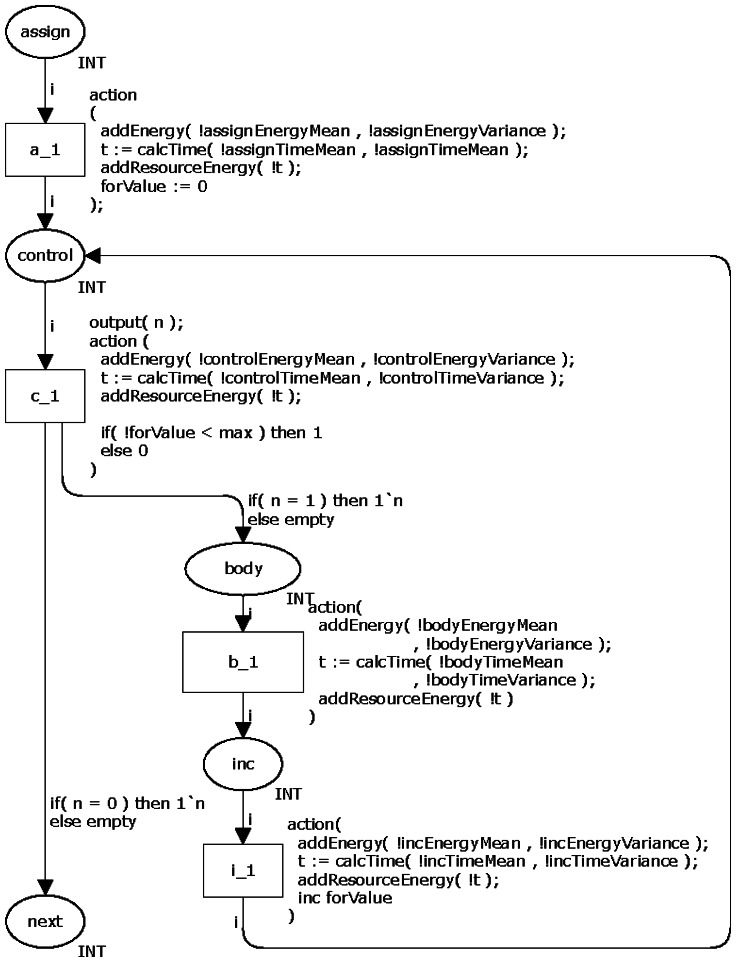
CPN model of the command *for*.

Figure 7 illustrates the proposed CPN model for representing the command *for*. The places *assign*, *control*, *inc* and *body* model by the initialization, control, step and body, respectively. The initialization is performed before the loop itself and occurs only once. The transition *c_1* decides the number of times (N) the loop must repeat based on a value set by the application developer. This model has dead transitions in the case a probability associated to the command is equal to zero.

#### 3.1.4. Calling Command: call

As mentioned in Section 2.2.1, the calling command call *α* is used to invoke a nesC command (*α*), to signal an event (*ε*) or to post a task (Ω). As a consequence, the proposed model has some particular elements when compared to the previous one. Firstly, the power consumption depends on the function α being invoked; post command does not follow this rule because it only tells to scheduler what task should run next. Secondly, in the case an event *ε* (e.g., sendDone()) must be signalised after executing the function α (e.g., send()), the event *ε* must be pushed into a function queue (described in Section 3.3); while a task must always be queued. Additionally, the execution of call may enable/disable a mote's resource (like radio or LED), which has an impact on the overall power consumption calculation.

**Figure 8.**
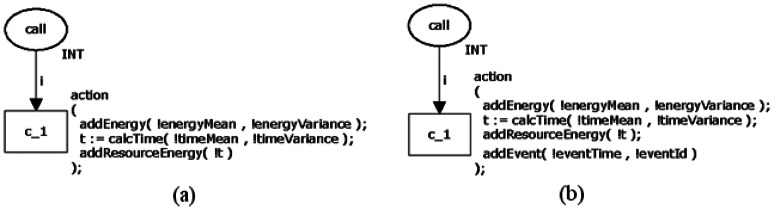
CPN models of the command *call*.

Figure 8 depicts two CPN models for representing the *call* command. First model (a) is very similar (with the same properties of the model) to one shown in Figure 3, whilst the second one (b) models the situation in which an event or task will be signalised after the completion of the command being invoked. In the last case, the function addFunction() push the function (to be signalised) into the aforementioned function queue and has two parameters: the time (*functionTime*) the task or event must execute and the function identifier (*functionId*).

#### 3.1.5. Event receive

**Figure 9.**
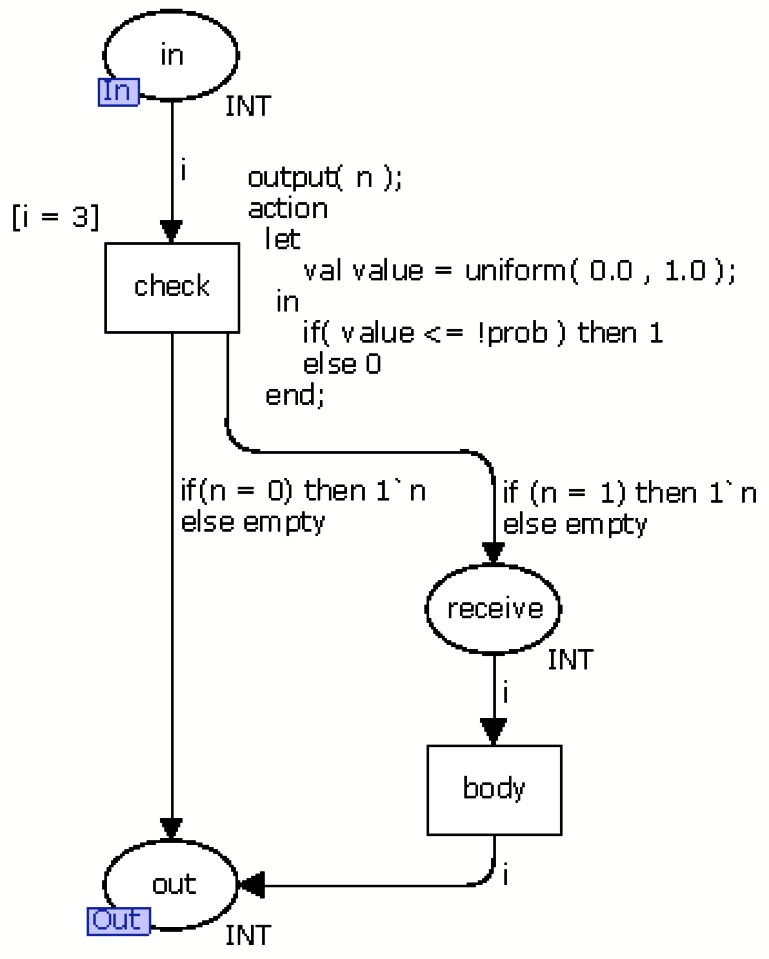
CPN model of the event *receive*.

Generally, the events in the nesC language represent responses to the execution of a command. For example, when an application executes the command *start* to enable a radio, it discovers if the radio is turned on through the event *startDone*. Another example is when the application collects a temperature. It executes the command *read* and receives a value through the event *readDone* when the temperature is collected. Additionally, there is an event, named *receive*, that depends on the occurrence of an external event. The event *receive* is triggered when a new message arrives in the sensor node. Unlike the previous events, this event has a probability associated that indicates the chance of its occurrence. Figure 9 presents the CPN model (called module *receive*) of this event.

The module *receive* has a transition, named *check*, to decides if it receives a message by generating a random value between 0 and 1 following the uniform distribution. If the generated value is less than or equal to the probability this event occurs, the token moves from place *in* to *receive*. Otherwise, token moves to *out*. Places *in* and *out* are input and output ports, respectively (see Section 2.1.1). The transition *body* models the command(s) executed in this event. The CPN model of the others events have the places (*in* and *out*) and the transition *body*.

### 3.2. Function Models

Prior to present what is a Function models, it is worth remembering that the implementation of a nesC application consists of a set of functions, named task, command and events (see Section 2.2.1), that are very similar to C functions. On the other hand, a function itself is a block of code containing a set of operators/statements such as assignments, loops and *if-then-else*. As presented in the previous section, these elements are modelled by the Basic models. Therefore, a Function model consists of a set of Basic models that are put together according to a proposed rule. In order to better illustrate this composition, consider the following nesC function (command):
command void Module.read(){SLEEP_TIME = 100; //assigncall Temp.read(); //invocation}

**Figure 10.**
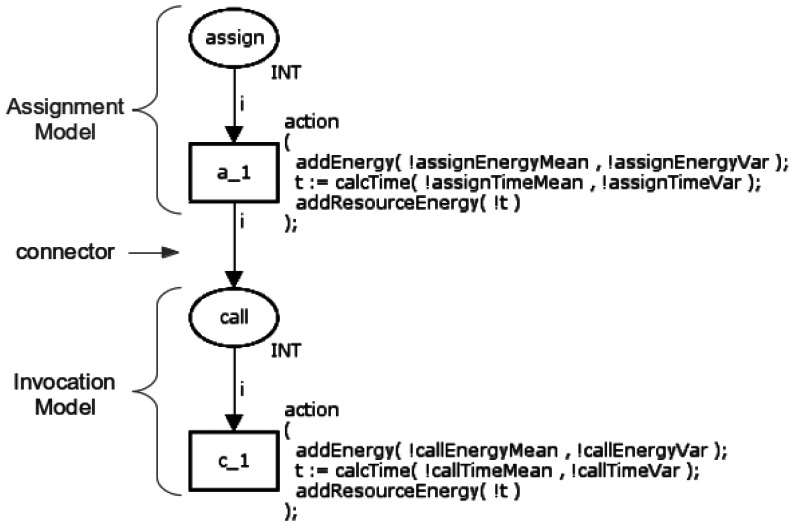
CPN Model of the function.

This function has two elements, assignment (=) and invocation (call), that are executed sequentially. Hence, its respective Function model is simply the composition of the Basic models of these two operators as shown in Figure 10.

The assignment operator is modelled through the place *assign* and the transition *a_1*, whilst the invocation operator is modelled by the place *call* and the transition *c_1* (see Section 3.1.1). These Basic models are merged according to the following rule: the transition from the first model (*a_1*) is connected (connector) to the place in the second model (*call*). This rule is used to compose all Basic models.

### 3.3. Application Models

To understand how the application model is obtained, it is important to observe that applications written in nesC are event-driven in such way that the order the application executes is only partially defined at compile time (see Section 2.2.1) due to the occurrence of tasks and events (e.g., the arrival of a message sent by another WSN node). Hence, a modelling element, named *scheduler*, has been introduced to explicitly treat with the occurrence of these tasks or events, and serves as the key element in the application models. These features make the application model generic, allowing represent any nesC application independent from the hardware platform.

**Figure 11.**
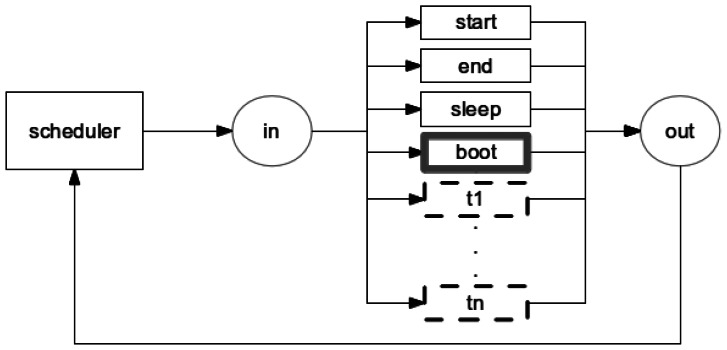
Schematic representation of the Application model.

Each application model is organized into CPN modules (see Section 2.1.1) and each module models nesC tasks, commands and events (see Section 2.2.1). Events that depends on the occurrence of an external factor (e.g., another WSN node sends a message), has a probability associated by the application developer that indicates the probability and time of this particular event occurs. Figure 11 shows a schematic view of the Application model.

The *scheduler* has the following basic elements: a function queue, five transitions (*scheduler*, *start*, *end, sleep*, *boot*) and two places (*in*, *out*). The tasks and events are stored in a queue and each one has associated an identifier and the time indicating when the task or event must occur. In this way, tasks or events are removed from the queue based on this time, e.g., the queued function with shortest time is the next to occur. In the case two different functions have the same time, one first queued is the next to be triggered.

The transition *scheduler* acts as an orchestrator that defines the next transition (or module) to be triggered according to the time associated to the task or event. Additionally, the task or event that is type AC will be selected by the transition *scheduler* and it can not preempt the execution SC. In other words, AC is executed like a SC (see Section 2.2.1).

At this point, it is worth observing that the event *receive* (see Section 3.1.5) is never putted in queue, as it depends on an external event (when a third-party message arrives in the mote). We solved this problem by allowing the *scheduler* to alternatively select an event from the queue and the event *receive*. Additionally, the radio should be enabled to run the module *receive*.

The places *in* and *out* are sockets and identify the beginning and the end of activities, respectively. The place *in* is also the starting point of the model, indicating that the first transition to be performed (the other transitions will be chosen by the transition *scheduler*). It worth observing that the combination of transition *scheduler*, that decides which activity should be performed, with the place *in*, starting point of the application model and beginning of activities, leads to a reversible model. In other words, the model will always return to the starting point of the model through the transition *scheduler*.

In the case the queue is empty (no function is ready to be triggered), the transition *sleep* is triggered indicating that the application does not need to execute any task. The transition *start* is executed only once, it initializes all variables and puts the first function in the function queue. The transition *end* indicates the end of the application execution and may be executed several times.

The transitions *boot*, *timer, t_1_*, and *t_n_* depend on the application being modelled. These transitions correspond to task or event implemented by the application and each transition serves as a bridge between the module *scheduler* and the respective function module. For example, the transitions *boot* and *timer* represents the respective *boot* and *timer* module. The transition *t_1_* and *t_n_* represents other function (task or event) implemented by the application.

### 3.4. Power-Related Functions

The CPN models introduced in the previous sections use three auxiliary functions that we have defined to compute power-related measurements: addEnergy(), calcTime() and addResourceEnergy(). Functions addEnergy()and calcTime() are responsible for calculating the power consumption and performance, respectively, of an operator modelled. On the other hand, function addResourceEnergy()is used to calculate the power consumption of the resource (radio and LEDs).

The pseudo-code (Due to the complexity of the syntax of CPN ML, we adopted a pseudo-code to facilitate the understanding of the function codes.) of the function addEnergy is presented in the following:

1Function addEnergy (real mean, real variance){
2 var energy = normal (mean, variance);
3 energy_app = (!energy_app) + energy;
4 return energy;
5}

The parameters mean and variance are used to calculate a random value following the normal distribution (variable energy in line 2) that represents the power consumption of a transition. It is worth observing that function normal() belongs to the CPN Tools library. The generated value is then added to the variable (energy_app) used to store the application's power consumption. Function calcTime is similar to addEnergy, but it generates a random time associated to each transition.

The proposed infrastructure supports many distribution probabilities, among them exponential, Erlang, polynomials, normal, and so on [27]. The system considered in this work, however, being an embedded system in which each mote is mainly devoted to few local non interfering activities, makes the system quite stable as can witnessed through the measures collected. Nevertheless, variation still exits, since the system is not deterministic. In this study, the normal probability distribution was the one that best fitted the measured data. Finally, the pseudo-code of function addResourceEnergy is shown in the following:

1function addResourceEnergy (real time) {
2 if (radioIsOn) {
3  var energy = radio_power * time;
4  energy_app = (!energy_app) + energy;
5 }
6 if (led0IsOn) {
7  var energy = led0_power * time;
8  energy_app = (!energy_app) + energy;
9 }
10 if (led1IsOn) {
11  var energy = led1_power * time;
12  energy_app = (!energy_app) + energy;
13 }
14 if (led2IsOn) {
15  var energy = led2_power * time;
16  energy_app = (!energy_app) + energy;
17 }
18}

This function calculates the power consumption of the resources (e.g., LEDs) available in the sensor node. In this particular case, the adopted mote (IRIS mote) has one radio and three LEDs. Each resource has associated a global variable that indicates if the resource is active (on) or not. In the case the resource is on, the power consumption due to the resource is calculated and added to the variable that stores the application's power consumption (energy_app). It is worth observing that as we do not know how long the resource is on, this function is associated to each transition present in the model.

### 3.5. Translator

In order to automatically generate the function and application models along with their power consumption evaluation, the *nesc2cpn* translator was implemented. The translator n*esc2cpn* generates the power consumption models from nesC programs and then interacts with the CPN Tools that simulates and yields power consumption metrics of these models.

**Figure 12.**
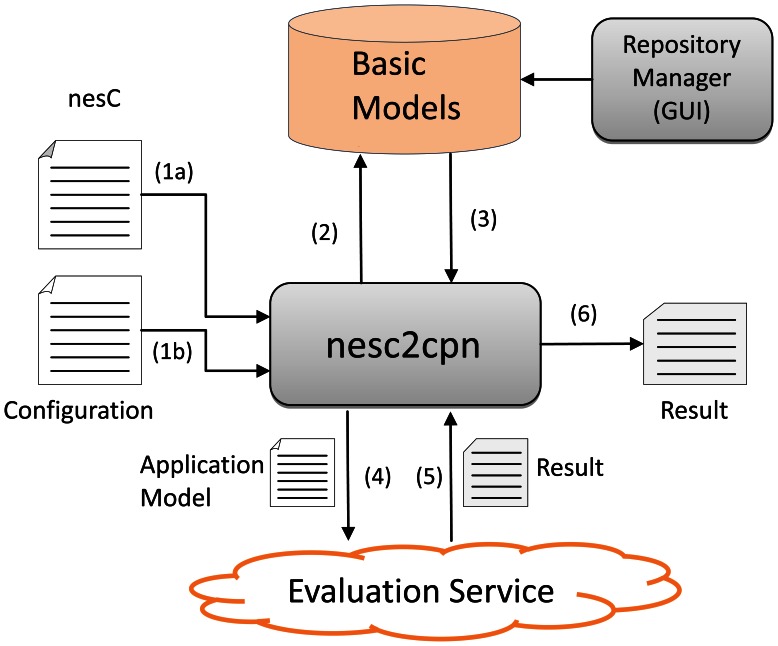
Overview of *nesc2cpn* translator.

According to Figure 12, the *nesc2cpn* receives a nesC program as input (1a) and some configurations parameters such as hardware platform, level of confidence and batch length (1b) (see Section 4.2.1). Next, it accesses the repository of CPN basic models (2-3) and generates the respective application model. The generated application model is then passed to the Evaluation Service (4) that simulates it in the Internet and yields the power consumption results (mean, variance and standard deviation) (5-6).

The *Evaluation Service* is a Web service (implemented in Java, available at http://ec2-23-22-219-74.compute-1.amazonaws.com:8080/EvaluationService/ws?wsdl) that has two functions, named evaluateApplication and getResult. Function evaluateApplication is used to evaluate a model asynchronously as the evaluation can be take hours or days. When this function is invoked, it saves the model (to be evaluated latter) and returns a *request id*. This *request id* is used to get the results of the evaluation by invoking the function getResult.

The *nesc2cpn* translator is an API (implemented in Java, available at https://github.com/sensor2model-group/nesc2cpn ), which provides three methods: evaluateSync, EvaluateAsync, getResult. The first two methods are used to generate and evaluate the CPN models in a synchronous or asynchronous way; method getResult is used to retrieve the results when the model in evaluates in a asynchronous way. Finally, the *Repository Manager* enable us to include and update the basic models in the repository, e.g., to add a basic model of a new hardware platform.

## 4. Experimental Evaluation

The goal of this experimental evaluation is to compare the power consumption of nesC applications using two different techniques: measurement, by executing the nesC applications in an actual mote; and simulation, using the CPN models of the nesC applications generated by the *nesc2cpn* translator.

Next sections present how the measurements were carried out using actual motes (Section 4.1) and the comparison between simulation and measurement results (Section 4.2).

### 4.1. Measurement Procedure

The nesC applications used in the both experiments were deployed in an IRIS (the datasheet of the IRIS mote is available at http://www.memsic.com/products.html) mote, connected to a MTS400 basic environment sensor board. These applications were compiled by nesC version 1.3.1, built on top of the TinyOS 2.1.1. An oscilloscope (Agilent DSO03202A) was used to measure the power consumption of the mote executing the applications. A PC was connected to the oscilloscope that captures the code snippet execution start and end times by monitoring a LED of the mote, which is turned on/off to signal the execution start/end. The PC runs a tool named AMALGHMA [28] that was responsible for calculating the power consumption.

In order to measure the power consumption of each nesC operator (see Section 2.2.1) and built-in nesC commands available in TinyOS (e.g., send, receive, read), it was created a generic application as shown in the following:

1implemention {
2
3 event void Boot.booted(){
4  call Timer.startPeriodic (100);
5 }
6
7 event void Timer.fired(){ //start power measurement
8  operator; //first time
9  operator; //second time
10  …
11  operator; //*N - 1* time
12  operator; //*N* time
13 } //stop power measurement
14}

To reduce the interference in the measurement process, this application does not enable any mote resource (e.g., radio and LED) and repeats the measuring until the average value achieves a specified error. The power consumption of a particular operator ( operator) is obtained by dividing the obtained measurement by the number of repetitions.

A similar approach was used to assess the power consumption of the mote's resources (e.g., radio and LEDs). However, in this case a simple application was executed with and without the particular resource active (on). The difference between both power consumption measurements is the resource's power consumption.

### 4.2. Application

As the focus of this paper is to evaluate the application, we have implemented five nesC applications and generated their respective CPN models using the nesc2cpn. These applications have the following characteristics: an application only containing nesC statements (see Section 3.1.1), more specifically this application (named *App1*) calculates the average of five numbers; an application that has one invoke to collect the temperature, which is a typical WSN application (named *App2*); an application that uses a selection statement (see Section 3.1.2) for aggregating sensed values (named *App3*); an application that calculates the average of five sensed values (similar to *App1*), but used together an iteration statement (named *App4*); and an application that collects the temperature and sends it to another sensor node (named *App5*).

We evaluated a small piece of code of *App1* to *App4* and an entire application was evaluated in *App5*. These applications serve to show how the tool and proposed model are flexible in such way that they can be used to evaluate a small piece of code or an entire application. Prior to present the evaluation itself, it is worth introducing the stopping criteria adopted in the simulations.

#### 4.2.1. Stopping Criteria

The simulation of proposed application models is steady-state and takes a long duration. In practice, it is necessary to define a criterion to stop the simulation. The batch means is one of the best solutions to solve this problem and is widely adopted.

The batch means divides the simulation parts (called batch) with similar sizes. Next, it computes the average of each batch and constructs a list (called batch means) with theses averages. We can determine the error of the simulation through batch means. The error is calculated with the following equation:

(1) $$E=\frac{t^{\left(k-,1,-\alpha \right)}\times s}{\sqrt{k}}$$

where *t* is the value of the T-Student distribution with degree of freedom *K-1* and level of confidence (defined by user) equal to *1-α*, *k* is the size of the batch means and *S* is the standard derivation of the batch means. We compare the error of the batch means (*E*) with a value defined by the user, called “max error”. The simulation stops when *E* is less than or equal to the max error.

#### 4.2.2. Result

The first nesC application (*App1*) is showed in the following:

1implemention {
2 int8_t mean = 0, a = 8, b = 16, c = 32, d = 64, e = 127;
3
4 event void Boot.booted(){
5  call Timer.startPeriodic (100);
6   }
7
8 event void Timer.fired(){ //start measure
9  mean = (a + b + c + d + e) / 5;
10 } //end measure
11}

This application would have only one assignment command (see Section 3.1.1). However, its power consumption would be equal to an application without any command. This occurs because as the variable will not used in the program (as the program has only one assignment), the compiler performs an optimization and discards this assignment in order to reduce the power consumption. Hence, the application *App1*, whose purpose is to evaluate the assignment command, also includes arithmetic operators (+ and /).

**Figure 13.**
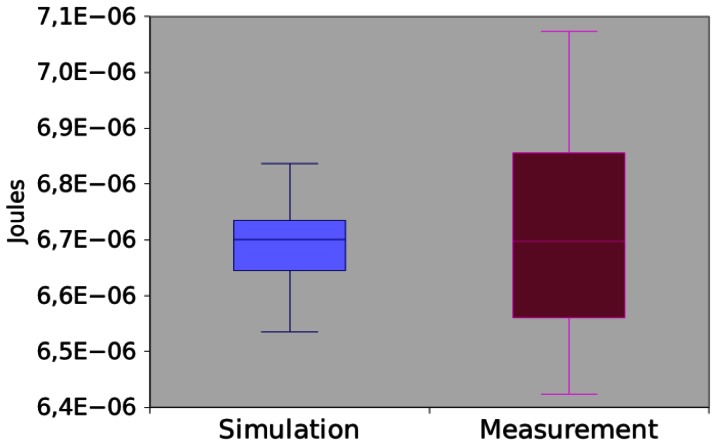
Power consumption of *App1*.

Figure 13 shows the power consumption of *App1* trough measurement and simulation. The mean difference between simulation and measurement is 0.35%. As the values are very close, we decided to check if those means are significantly different. By applying the hypothesis testing, it returns a p-value equal to 0.520920. Hence, we concluded that there is no sufficient evidence to reject that simulation and measurement returned different values.

The second nesC application (*App2*) simply collects a temperature:

1…
2void readTemperature(){ //start measure
3 call Temp.read();
4}
5
6event void Tem.readDone(error_t err, float value){
7 // do nothing
8} //end measure
9…

**Figure 14.**
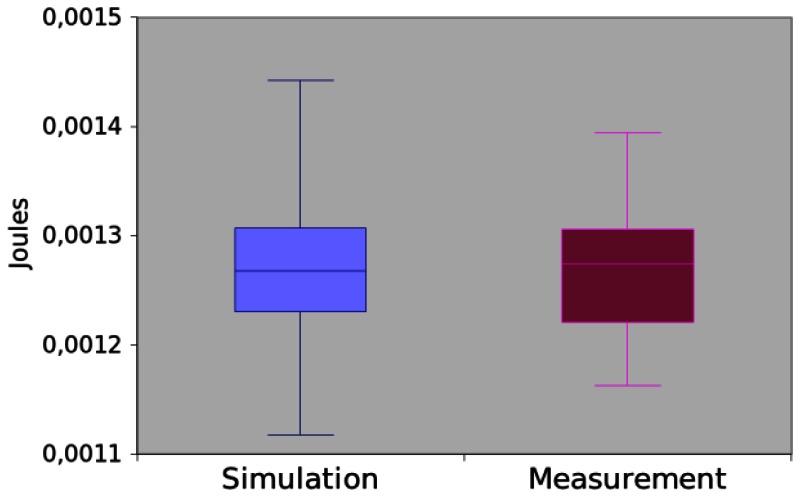
Power consumption of *App2*.

The initial code of this application is very similar to *App1. App2* invokes the command read (3) to collect a temperature and the event readDone is triggered when the reading operation terminates, *i.e.*, the sensor obtained the temperature. Figure 14 illustrates that the results obtained by measurement and simulation are very similar and they have a difference around 0.19%. Additionally, we confirmed that they are similar as the hypothesis testing returns p-value equal to 0.8276.

*App3* has a function that uses three variables for aggregating data along with a selection statement (*if-then-else*):

1…
2void aggregate(int8_t value){ //start measure
3 total = total + value;
4 size = size + 1;
5
6 if (size == 5){ //@0.20
7
8  mean = total / 5;
9  total = 0;
10  size = 0;
11 }
12} //end measure…

The declaration of variables and boot code (1) are similar to *App1*. The function aggregate(), as commonly found in WSN applications, makes a calculation (e.g., average) over obtained measures. The statement *if-then-else* has associated a probability of 20% (//@0.20) because it is executes once one in five.

**Figure 15.**
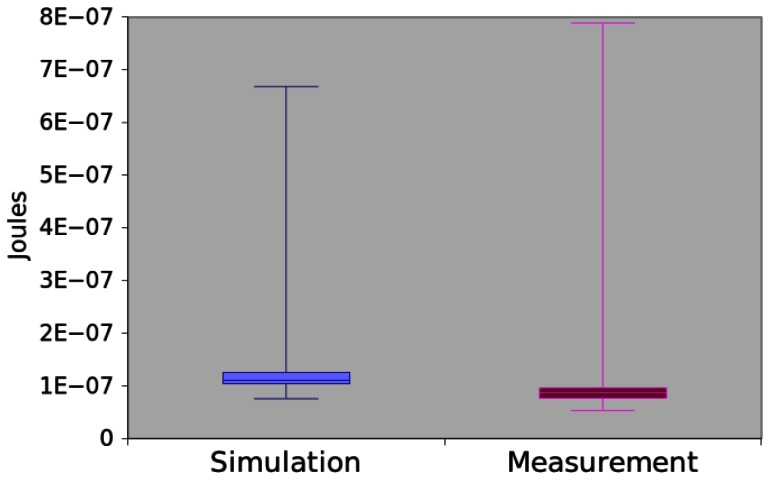
Power consumption of *App3*.

The results obtained by simulation and measurement are illustrated in the Figure 15. The mean difference between two methods was of 23.30%. Again, we apply the hypothesis testing to them (generated p-values equal to 0.115351), which shows that there is not sufficient evidence to assert that the methods return different data.

The fourth application, namely *App4*, is shown in the following:

1…
2void mean(){ //start measure
3 total = 0;
4  for(i = 0; i < 5; i++){ //@5.0
5  total = total + value [i];
6 }
7 mean = total / 5;
8} //end measure

**Figure 16.**
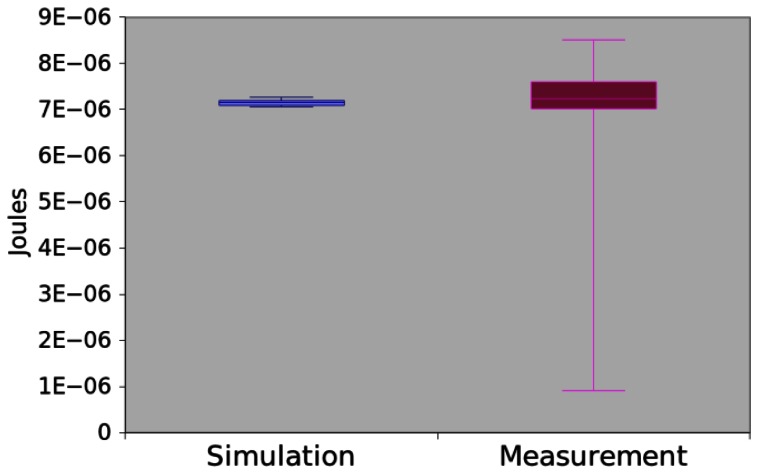
Power consumption of App4.

Similarly to *App1*, this program calculates the average of five numbers, but it uses an iteration statement (*for*) to sum them (4-6). Figure 16 shows the power consumption obtained through measurement and simulation. In this case, the mean difference is around 3.09% and the hypothesis testing returned a p-value equal to 0.072628. Hence, there is no evidence that simulation and measurement values are different.

Finally, the last application (*App5*) collects the temperature and sends it to another sensor node as shown in the following:

1implemention{
2 message_t pckt; msg_t* msg;
3
4 event void Boot.booted(){
5
6  msg = (msg_t*) call Packet.getPayload (&pckt, size (msg_t));
7  call RadioControl.start();
8 }
9
10 event void RadioControl.startDone(error_t e){
11  call Timer.startOneShot (100);
12 }
13
14 event void Timer.fired(){
15
16  call Temp.read();
17 }
18
19 event void Temp.readDone(error_t e, float value){
20  msg->error = e;
21  msg ->value = value;
22  call RadioSender.send (BASE_STATION, &pckt, 28);
23 }
24
25
26 event void RadioSender.sendDone(error_t e, message_t p){
27  call Timer.startOneShot (100);
28   }
29  }

The behaviour of this application consists of turning on the radio in the boot (4–7). When the radio is already started, the application collects a temperature (13–15) after 100 milliseconds (9–11). Next, the application creates a message and sends it to the base station (17–21). This behaviour is repeated each 100 milliseconds (23–25) in a loop. shows the *scheduler* (see Section 3.3) of *App5*.

**Figure 18.**
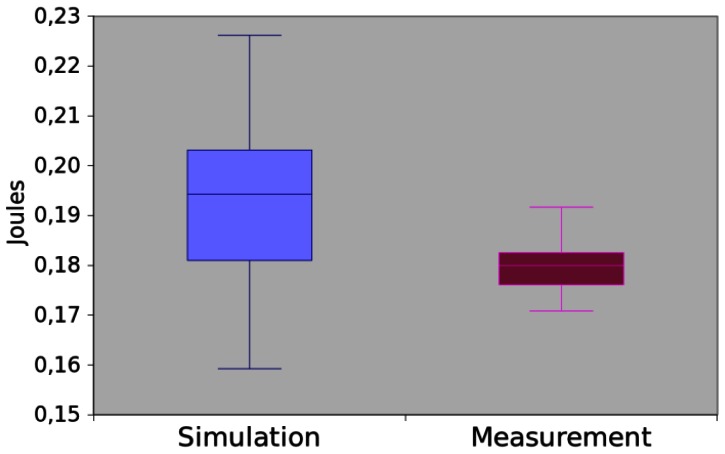
Power consumption of *App5*.

The results obtained by both methods are depicted in Figure 18. The mean difference between them was of 6.71%. The hypothesis testing returns the p-value equal to 0.083238. Hence, we can assume that, for this experiment, both methods return the same values.

The results of the evaluation of these five applications shows that the proposed approach is flexible by allowing the evaluation of either an entire application or just a piece of code (function). It is also possible to observe that the results obtained by measurement and simulation are very close in all cases. Additionally, this test also shows that for more complex codes (which likely consumes more power) and for small applications (which likely consume less power) there is no difference between both methods.

## 5. Related Work

In this section, we present and compare existing approaches for evaluating the power consumption of WSN applications. These approaches have been grouped considering the artefact available for the evaluation (source code, behaviour/states description) and evaluation technique adopted (measurement, simulation and analytical modelling).

### 5.1. Measurement

Hiltunen *et al.* [2] evaluated the performance and power consumption of the POCOBOS middleware through measurement in Imote2. In particular, their objective was to show the impact on the middleware in the performance and power consumption.

Lajara *et al.* [3] also used the measurement technique, but focused on comparing the power consumption of WSN operating systems (e.g., TinyOS, Contiki and others). In order to perform the comparison, two motes were used (Tmote Sky and MICAz) and four different applications, representing common task (e.g., read a temperature and send this value).

Chan *et al.* [1] measured the power consumption of different encryption algorithms (e.g., RC5, RSA) in Mica2. They created an application that encrypts a message and send it. The resulting power consumption is obtained by adding the CPU's power consumption (to execute the algorithm) and the power consumption due to the message transmission (to send the encrypted message).

In all cases, the aforementioned approaches need to assembly an environment to perform the measurements: (1) the application, middleware or operating system code (2) the WSN motes (e.g., IRIS, MICAz) to deploy the programming code; (3) the power supply (e.g., Icel Manaus PS-1500) to maintain the constant voltage of the WSN mote; (4) one oscilloscope (e.g., Agilent DSO3102A) to the measurement data; and (5) one program that collects data from the oscilloscope (e.g., AMALGHMA). One initial limitation of measurement approaches is the setting-up of the environment that is composed by several elements. Additionally, WSNs are usually composed by thousands of nodes and it is usually unfeasible to measure the power consumption of individual nodes.

### 5.2. Simulation

Simulation approaches mainly focus on defining and simulating models of architectures (hardware platform), operating systems and applications. AVRORA [29] provides a cycle-accurate model of the MICA mote family and has been used to evaluate the power consumption of MICA and MICA2 hardware platforms.

While some simulations are related to a specific mote or mote family, Somov [30] presents a simulation framework to estimate power consumption of WSN applications for arbitrary hardware platforms. This framework describes the possible states of each component (e.g., memory and radio) separately and each state has the energy consumption and performance associated. A set of these components represents a particular hardware platform.

The need of both evaluating new operating systems and testing real-code applications before their deployment motivated the appearance of operating system emulation environments [31]. A well-known example is the TOSSIM [32] simulator for TinyOS. Its main propose is to execute TinyOS applications without their deployment in actual WSN nodes. TOSSIM ignores some hardware behaviours like interruptions of the CPU.

Unlike the aforementioned approaches that simulate directly the programming code, existing researches also concentrate on defining behavioural models of WSNs. Shareef and Zhu [33] use Deterministic and Stochastic Petri Nets (DSPNs) to model the behaviour of IMote2's CPU and radio. Each place represents a state of the processor or the radio. The number of tokens present in each place at the end of simulation represents the time spent (in percentage terms) in that state. To find out the consumption of each state, it multiplies the amount of tokens by the average power consumption of the respective state. NS-2 [34] and OPNET [35] are well-known simulators that may be used to evaluate the power consumption of WSNs.

Most of these applications are strongly coupled to a particular hardware platform, which makes difficult the adoption of the same approach for different WSN motes. In particular, our solution facilitates the evaluation of new platforms, as the presented models are generic and only need to be configured to a particular mote. Another advantage of the proposed approach is the generation of power consumption results from a programming language code in an automatic way.

### 5.3. Analytical Modelling

Current approaches that use analytical modelling focus on the evaluation of WSN networks and not in WSN applications: Shinghal *et al.* [18] estimated the lifetime of particular WSN motes developed by them; Rusli *at al.* [36] evaluated the performance of the Opportunity Routing (OR) protocol [37]; Manjeshwar *et al.* [38] modelled, evaluated and compared the energy consumption of two protocols, namely LEACH [39,40] and APTEEN [41]); Sahota *et al.* [42] proposed and evaluated a new MAC protocol name DS-MAC [43]; and Cano *et al.* [44] verify the benefits (overhearing and collision) of Low Power Listening (LPL) [45] of MAC protocols.

## 6. Conclusions and Future Work

This paper presented an approach for evaluating the power consumption of WSN applications through simulation. The proposed approach consists of a set CPN models that represents the power consumption of nesC operators, which are assembled together to model the power consumption of the whole application. Basic in this strategy is the development and implementation of the *nesc2cpn* translator that is responsible for both generating the CPN models and evaluating their power consumption by interacting with the CPN Tools.

The main contribution of this paper is the proposition of an approach that automatically generates the power consumption of a WSN application straight from its nesC code. By composing the proposed CPN models, the translator is able to generate function and application models that allow a fine and coarse grained power consumption evaluation of WSN applications. The proposed models and the translator were evaluated in such way that the power consumption results obtained through simulating the generated models and actual measurement were very similar and it was not possible to make distinction between them.

We have now started to solve the limitations of the model (e.g., asynchronous codes) and to extend the proposed solution in order to allow the power consumption evaluation of wireless sensor networks (e.g., thousands of WSN nodes, network protocols and lower power listening) using the generated application models. Due to the possible increase in the size of simulation models, the implemented tools are now being migrated to a cloud computing environment with a greater processing power. Finally, a nesC editor is now being developed and integrated into the proposed translator to allow a power aware development of WSN applications.

## Figures and Tables

**Figure 17.**
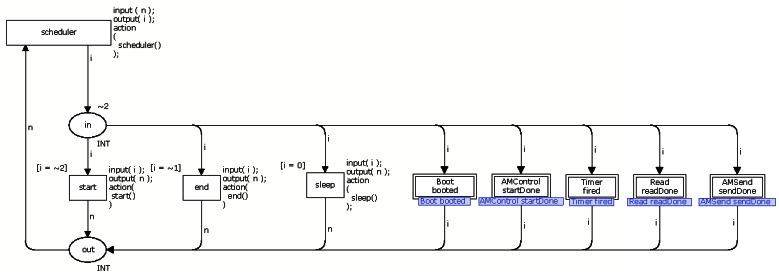
module *scheduler* of *App5*.
